# Supplementary figures and images for: Heterogeneous taxonomic resolution of cytochrome b gene identification of bats from Argentina: Implications for field studies
Source: PLoS One. 2020 Dec 31;15(12):e0244750. doi: 10.1371/journal.pone.0244750 (PMC7775095; doi:10.1371/journal.pone.0244750)

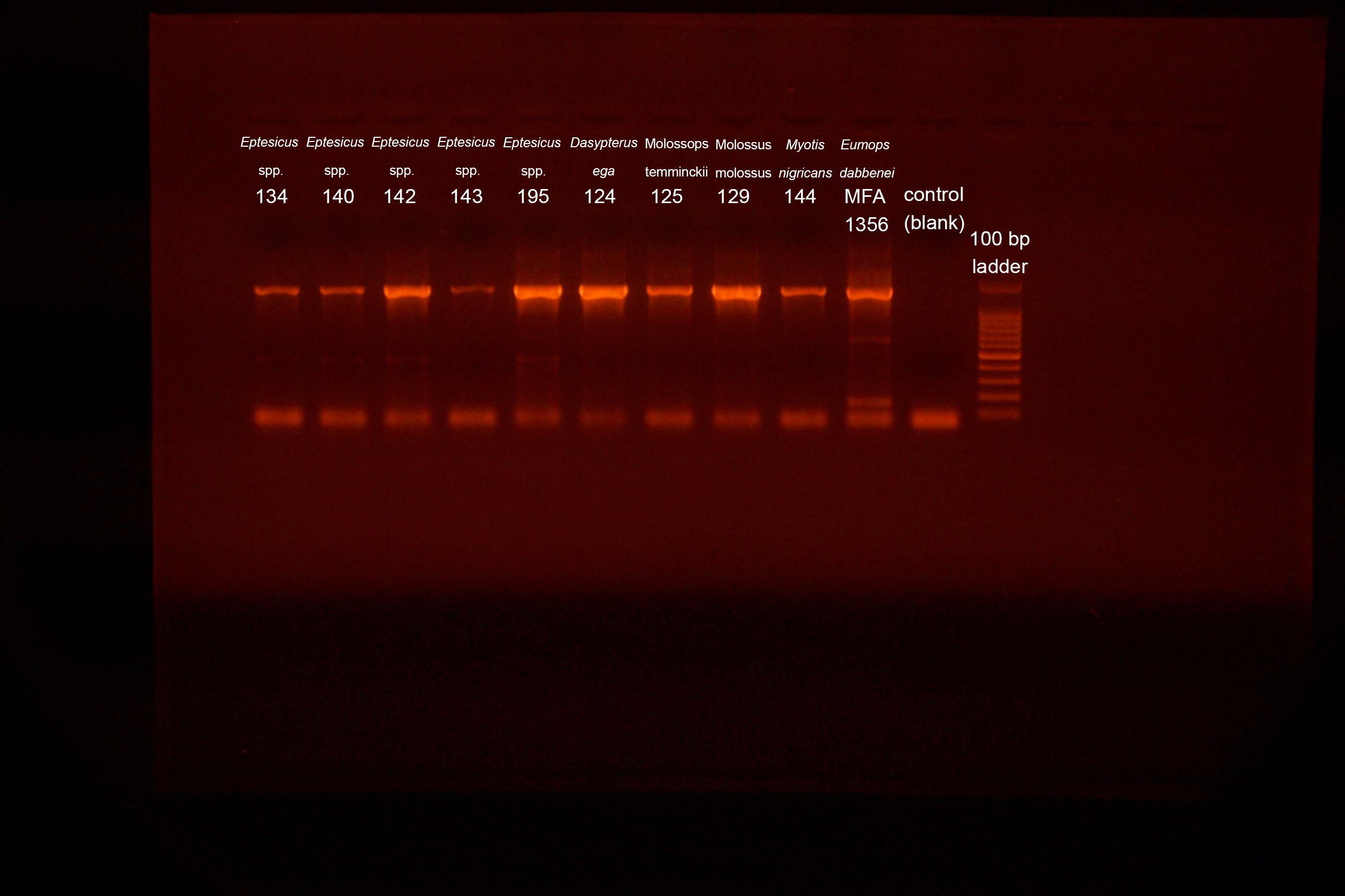

Supplement: S1 Fig — The combination of primers Bat 05A and Bat-Ep successfully amplified the Cytb gene and flanking regions in all assayed species, yielding a PCR product of ca 1330 bp. From left to right, PCR products were obtained in Eptesicus (lanes 1–5), Dasypterus (lane 6), Molossops (lane 7), Molossus (lane 8), Myotis (lane 9), and Eumops (lane 10). Lane 11 shows the PCR negative control, while lane 12 corresponds to the molecular-weight size marker. (JPG) [file pone.0244750.s004.jpg]

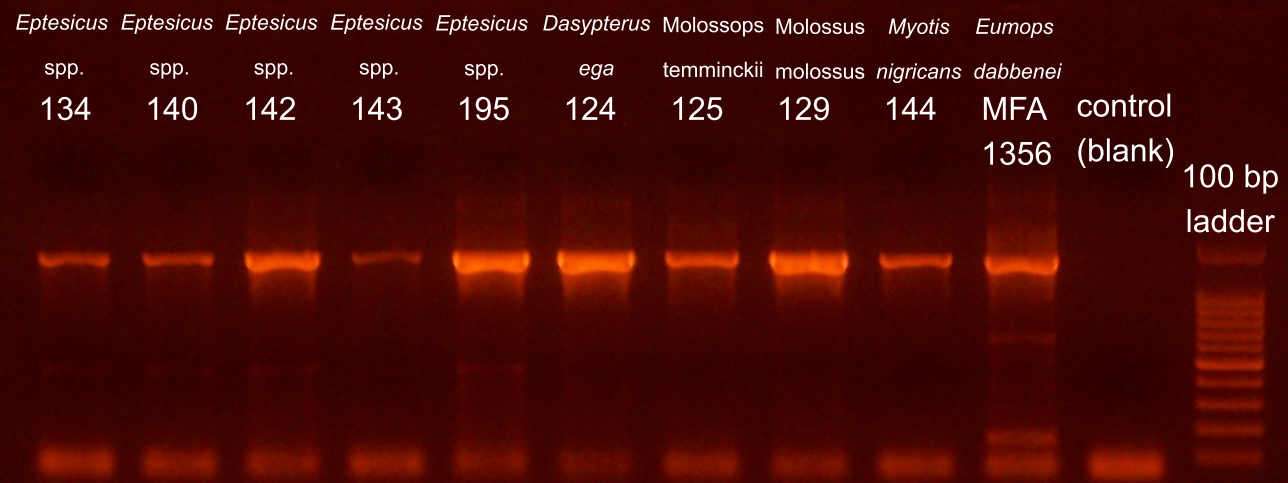

Supplement: S1 Raw image — Original and uncropped image underlying gel results. (PDF) [file pone.0244750.s005.pdf]
